# Metal halide perovskites for photocatalytic applications: opportunities and challenges

**DOI:** 10.1093/nsr/nwaf376

**Published:** 2025-09-09

**Authors:** Chunhua Wang, Ning Han, Yang Ding, Yun Hau Ng, Bao-Lian Su

**Affiliations:** Department of Electrical Engineering and Computer Science, University of Michigan, USA; Department of Electrical and Computer Engineering, University of Toronto, Canada; College of Materials and Environmental Engineering, Hangzhou Dianzi University, China; School of Energy and Environment, City University of Hong Kong, China; Chemical Engineering Program, Physical Science and Engineering (PSE) Division, King Abdullah University of Science and Technology (KAUST), Saudi Arabia; Laboratory of Inorganic Materials Chemistry (CMI), University of Namur, Belgium; State Key Laboratory of Advanced Technology for Materials Synthesis and Processing, Wuhan University of Technology, China

## Abstract

This work summarizes recent progress in metal halide perovskites for photocatalytic applications and pinpoints the critical challenges that currently limit their practical deployment.

Photocatalysis technology is regarded as a sustainable approach to addressing global challenges in clean energy production and environmental remediation [1,2]. Over the past decades, a wide range of photocatalysts with diverse structures, properties, and synthetic strategies have been developed. However, the performance of many of these materials remains limited by inefficient light absorption and poor charge separation [3,4]. Thus, exploring innovative semiconductor materials has always been a hot subject. Recently, metal halide perovskites (MHPs) have emerged as promising photocatalysts owing to their remarkable optoelectronic properties and low-temperature solution-synthesis, enabling their use in diverse photocatalytic reactions [5]. Despite great promise, several challenges still hinder the practical application of MHP-based photocatalysis.

In this Perspective, we first highlight the key physicochemical properties of MHPs that underpin their photocatalytic performance. Then, we summarize recent advances across major reaction systems and identify critical bottlenecks. Finally, we outline strategies to overcome current limitations and propose future directions for advancing MHP-based photocatalysis toward real-world applications.

MHPs can be categorized into several types based on their structural characteristics, including ABX_3_, A_2_B(I)B(III)X_6_, A_2_B(IV)X_6_, and A_3_B(III)_2_X_9_. They can be synthesized through various solution-based methods, such as antisolvent precipitation, hot injection, spin coating, and solvothermal approaches. These methods typically operate at low temperatures without requiring high vacuum or expensive infrastructure, making them more scalable and cost-effective than conventional semiconductor photocatalysts. Moreover, MHPs are generally less expensive than many noble-metal–based photocatalysts. Their excellent photocatalytic performance stems from several distinctive attributes (Fig. 1) [3,5]: (1) Superior light-harvesting capability: MHPs exhibit strong absorption across the visible and near-infrared spectrum, with absorption coefficients exceeding 10^5^ cm^−1^. This characteristic enables efficient utilization of sunlight and high photocatalytic activity even under low light intensities. (2) Long carrier lifetime and diffusion length: Carrier lifetimes of MHPs range from nanoseconds to microseconds, coupled with diffusion lengths up to hundreds of micrometers, facilitating efficient charge separation and transport. This minimizes charge recombination, which is critical for photocatalytic performance, especially in multi-electron reactions such as CO_2_ reduction. (3) Defect tolerance: Unlike many traditional semiconductors, MHPs are highly tolerant to structural defects. Their electronic configurations favor shallow trap states rather than deep recombination centers, thus reducing non-radiative recombination and preserving carrier lifetimes. (4) Tunable bandgap: The bandgap of MHPs can be finely tuned from ∼1.2 to 3.6 eV, allowing for precise adjustments to optoelectronic and physicochemical properties and enabling optimization for various redox reactions with different energy requirements. (5) Structural and interfacial versatility: MHPs exist in diverse dimensions (3D, 2D, 1D, and 0D). Consequently, the optoelectrical and catalytic properties of MHPs can be easily regulated. Moreover, their compatibility with a wide range of cocatalysts enables synergistic enhancement of charge separation and surface photoredox reactions.

**Figure 1.**
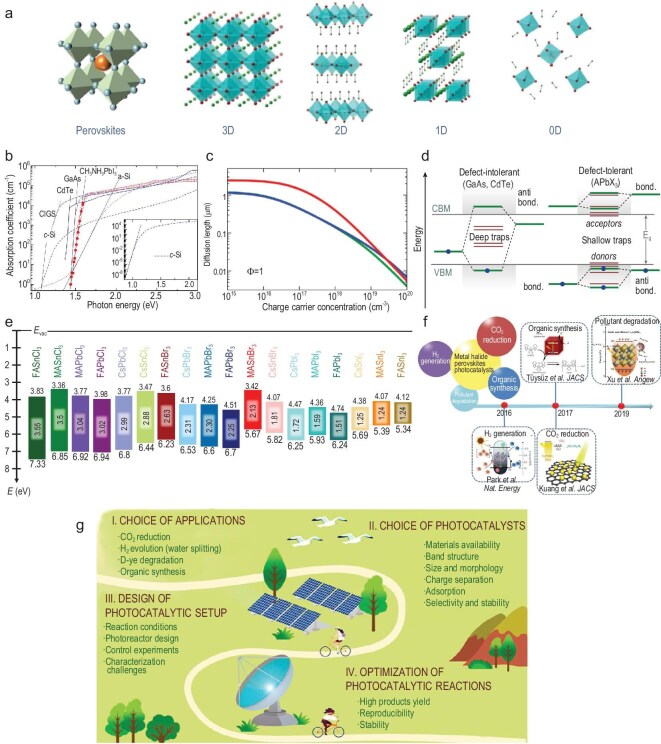
(a) Schematic illustration of MHPs with different dimensionalities. Reproduced with permission [7]. Copyright 2022, The Author(s), published by Springer Nature. (b) Comparison of the absorption coefficient of MAPbI_3_ material with traditional semiconductors. Reproduced with permission [8]. Copyright 2014, American Chemical Society. (c) An example of experimental determination of the diffusion length of MAPbI_3_. Reproduced with permission [9]. Copyright 2013, Wiley-VCH. (d) Comparison of the defect-tolerant property of MHPs with traditional defect-intolerant materials. Reproduced with permission [10]. Copyright 2020, The Royal Society of Chemistry. (e) The calculated energy levels of a series of MHP materials. Reproduced with permission [11]. Copyright 2019, The Author(s), published by Springer Nature. (f) Illustrating the photocatalytic applications over MHP materials. Reproduced with permission [12]. Copyright 2020, Science Press and Dalian Institute of Chemical Physics, Chinese Academy of Sciences, published by Elsevier and Science Press. (g) A summary of the issues that need to be taken into consideration for MHP-based photocatalysis. Reproduced with permission [13]. Copyright 2022, American Chemical Society.

To date, MHP-based photocatalysts have been explored for a variety of reaction systems (Fig. 1) [3]. The general mechanism involves photon absorption, charge generation, carrier separation, and subsequent surface redox reactions. Upon light irradiation, MHPs absorb photons to generate electron-hole pairs. These photogenerated charge carriers are then separated and migrate to the catalyst surface to participate in photoredox reactions, where electrons drive reduction reactions such as reducing protons to H_2_ and CO_2_ to form CO/CH_4_, while holes promote oxidation reactions such as oxidizing water to O_2_ or organic substrates to value-added products. Major applications include: (1) H_2_ evolution: Given that MHP materials are prone to decomposition in water, photocatalytic splitting of HX (X = I, Br) into H_2_ via a precipitation-solubility equilibrium strategy is the most common system. However, X^−^ acts as a sacrificial agent which is preferentially oxidized to X_3_^−^ over H_2_O oxidation during the reaction processes [5]. Consequently, additional reductants (e.g. H_3_PO_2_) are required to regenerate X^−^ from X_3_^−^, increasing costs and reducing the overall value of the reaction. Moreover, the strong acidity of HX limits the selection of cocatalysts, as some may be unstable under such conditions. (2) CO_2_ reduction: MHPs have shown notable advancements in photocatalytic CO_2_ reduction to value-added chemicals. Three main factors influence the selectivity, including active sites, cocatalysts, and Gibbs free energy changes. The excellent light absorption and favorable conduction band position enable effective CO_2_ reduction with high selectivity. However, the photocatalytic activity in most studies remains modest (typically <100 μmol g^−1^ h^−1^). Furthermore, the most reported products are CO and CH_4_, and achieving multi-carbon products over MHP-based photocatalysts is still challenging [3,6]. Furthermore, most CO_2_ photoreduction reactions are performed in organic solvents (e.g. ethyl acetate), which may decompose into carbon-containing species, affecting the assessment and quantification of CO_2_-derived products [6]. (3) Organic synthesis: Leveraging tunable redox potentials and strong visible-light absorption, MHPs have demonstrated promise in promoting light-driven organic transformations. However, the scope of reported reactions remains limited to simple transformations (e.g. benzyl alcohol or toluene oxidation) [5]. Expanding the reaction systems to more complex, industrially relevant processes is an important next step. (4) Pollutant degradation: Owing to their relatively high conduction band positions, MHPs possess sufficient redox ability, particularly for the degradation of organic pollutants and dyes. Generally, the degradation efficiency is influenced by several structural features of the pollutant molecules, including electron density and functional groups, aromaticity and conjugation, steric hindrance, and bond dissociation energies, as well as interactions with MHP surface terminations and trap states. However, since MHPs are generally sensitive to water, most studies are performed in alcoholic media (e.g. ethanol), limiting their practical applications in wastewater treatment [3]. Additionally, the potential leaching of toxic elements (e.g. Pb) raises additional environmental concerns [4], where the breakdown of these photocatalysts could lead to further pollution. Therefore, future research should focus on lead-free alternatives and evaluate performance in realistic aqueous environments.

To transition MHP-based photocatalysts from the laboratory to practical applications, several key challenges must be addressed. First, unsatisfactory performance. Most MHPs still show suboptimal photocatalytic activity. Several strategies, including cocatalyst loading, heterostructure construction, morphology modulation, and defect engineering, have been employed to improve the photocatalytic performance of MHPs [6]. Note that although recent studies suggest that certain types of low-concentration defects can enhance photoactivity, precisely controlling the types and concentrations of these defects remains challenging. Computational methods such as DFT are widely used tools to study the key aspects of the photocatalytic process, such as Gibbs free energy, product selectivity, and surface reactions. Second, stability issues. MHPs are prone to degradation under moisture, heat, and light, particularly in aqueous media. Their instability under operational conditions is a significant concern, as degradation can diminish photocatalytic performance and lifetime. To improve the stability of MHPs, some strategies have been recently developed, including (1) establishing a dynamic equilibrium by placing MHPs in saturated haloacid solutions, (2) developing useful physical protection strategies such as designing core-shell structures, crafting functional layers, and constructing encapsulation layers via photoelectrochemical systems to screen the MHPs from water molecules, and (3) exploring water-stable MHP materials [5]. Third, toxicity concerns. Many high-performance MHPs contain Pb elements, which pose environmental and health risks. Developing low-toxicity alternatives such as Sn- or Sb-based compounds is critical for sustainable applications [3]. Fourth, reaction mechanisms. A comprehensive understanding of the underlying mechanisms governing photocatalytic processes (charge transfer, surface reactions, and product selectivity) in MHPs is still lacking, yet this is critical for designing more efficient materials and optimizing photocatalytic systems [3,6]. Further research needs to focus on investigating the interactions between charge carriers and photocatalytic reactions, as well as elucidating the involved reaction pathways, both experimentally and theoretically. Finally, scalability and integration. Although the synthetic versatility of MHPs can be observed, achieving high-quality large-scale production remains challenging. There is a need for scalable, low-cost, and environmentally friendly synthesis methods [3,5]. Moreover, effective integration of MHPs into practical photocatalytic devices such as tandem systems is critical for real-world applications [14].
